# Influence of PCBM Nanocrystals on the Donor-Acceptor Polymer Ultraviolet Phototransistors

**DOI:** 10.3390/nano14211748

**Published:** 2024-10-30

**Authors:** Hong Zhu, Quanhua Chen, Lijian Chen, Rozalina Zakaria, Min-Su Park, Chee Leong Tan, Li Zhu, Yong Xu

**Affiliations:** 1 College of Integrated Circuit Science and Engineering, Nanjing University of Posts and Telecommunications, Nanjing 210023, China; 2020020116@njupt.edu.cn (H.Z.); 2021020307@njupt.edu.cn (Q.C.); 2020020217@njupt.edu.cn (L.C.); cheelong@njupt.edu.cn (C.L.T.); 2 Photonic Research Centre, University of Malaya, Kuala Lumpur 50630, Malaysia; rozalina@um.edu.my; 3 Department of Electronics Engineering, Dong-A University, Busan 49315, Republic of Korea; mpark@dau.ac.kr; 4 Guangdong Greater Bay Area Institute of Integrated Circuit and System, Guangzhou 510535, China

**Keywords:** polymer phototransistor, PCBM, nanocrystal, UV detection, band engineering

## Abstract

Organic phototransistors, renowned for their exceptional biocompatibility, hold promise in phototherapy for tracking the efficacy of photosensitive drugs within treatment areas. Nevertheless, it has been found that organic semiconductors are less effective in detecting ultraviolet (UV) light because of their narrow bandgap. Here, we show that UV photodetection in phototransistors using donor-acceptor (D-A) polymer semiconductors can be significantly enhanced by incorporating PCBM nanocrystals. This integration results in a band mismatch between the nanocrystals and the D-A polymer at the interface. These nanocrystals also demonstrate a notable capability of modulating threshold voltage under UV light. The devices incorporating nanocrystals exhibit a photoresponsivity of 0.16 A/W, surpassing the photoresponsivity of the devices without nanocrystals by 50%. The specific detection rate of devices with nanocrystals is around 2.00 × 10^10^ Jones, which is twice as high as that of devices without nanocrystals. The presented findings offer a potential avenue to improve the efficiency of polymer phototransistors for UV detection.

## 1. Introduction

UV radiation plays a crucial role in various biomedical areas, with applications ranging from UV-based skin therapies to the intricate analysis of DNA and proteins [1,2,3,4,5,6,7]. UV light detectors require high sensitivity and flexibility to deal with different situations in biomedicine areas effectively [8,9]. Organic UV phototransistors, distinguished by their organic semiconductor composition, offer exceptional flexibility, lightness, and biocompatibility, making them increasingly sought-after in various biomedical fields [10,11,12].

In recent years, the main development of organic phototransistor has been focused on the mobility and stability improvement of the material, such as poly[9,90-dioctyl-fluorene-2,7-diyl]-copoly[diphenylp-tolyl-amine-4,40-diyl] (BFE) [10], poly(9,9-di-n-octylfuorenyl-2,7-diyl) (PFO) [11], and poly[(9,9-dioctylfluorenyl2,7-dial)-alt-co-(bithiophene)] (F8T2) [12]. Within the spectrum of organic semiconductors, D-A polymer poly[(bithiophene)-alternate-(2,5-di(2-octyl-dodecyl)-3,6-di(thienyl)-pyrrolyl pyrrolidone)] (DPPT-TT) stands out for its exceptional potential, e.g., mobility as high as 0.6 cm^2^V⁻^1^s⁻^1^ and notable stability [13]. Unfortunately, a narrow bandgap limits UV sensitivity due to low quantum conversion efficiency in D-A polymers [14,15]. Therefore, fine-tuning the energy band structure is required to improve the detection performance.

In this work, the organic semiconductor selected was the D-A polymer of DPPT-TT. PCBM nanocrystals were used to modify its band structure. This decision was primarily driven by their efficient UV photon absorption capability, which has the potential to enhance both responsivity and efficiency. Simultaneously, the DPPT-TT and PCBM blend can form thin films using simple solution-based processes. Phototransistors using DPPT-TT:PCBM with a weight ratio of 1:0.2 exhibited an impressive photoresponsivity of 0.16 A/W, marking a 50% increase over the devices without nanocrystals. In addition, the maximum specific detection rate of those phototransistors with nanocrystals reached approximately 2.00 × 10^10^ Jones at a gate voltage of –20 V, doubling that of bare DPPT-TT phototransistors. Concurrently, the negative threshold voltage shift in devices with PCBM nanocrystals is due to the “floating gate” (or photo-doping) effect of the nanocrystals. Therefore, this work provides a solution for optimizing polymer UV phototransistors.

## 2. Experimental Section

**Figure 1:**
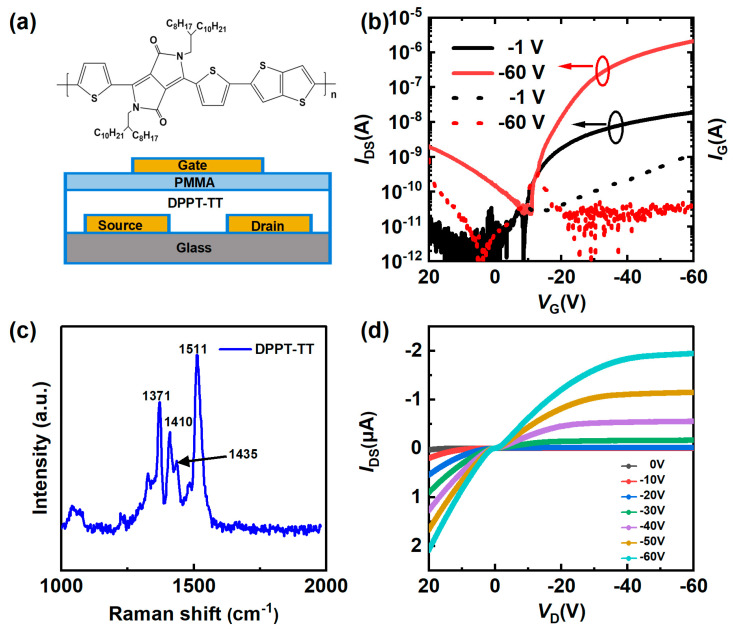
(**a**) Molecular structure of DPPT-TT and device structure. (**b**) Typical transfer characteristics of the phototransistor operating in the linear and saturation regimes. (**c**) Raman spectrum of DPPT-TT. (**d**) Typical output characteristics of the phototransistor.

The device architecture of the top-gate, bottom-contact organic phototransistor, and the molecular structure of DPPT-TT are depicted in Figure 1a. Eagle glass was first subjected to a cleaning procedure consisting of sequential ultrasonic immersions in alcohol, deionized water, and alcohol for 15 min each. Then, a 10 nm nickel (Ni) adhesion layer and 20 nm gold (Au) source-drain electrodes were deposited on the glass substrate by thermal evaporation using a shadow mask. The channel width (*W*) was 1200 μm, while the channel length (*L*) ranged from 150 μm to 500 μm. UV Ozone was used to clean the substrates to enhance their hydrophobic properties. Subsequently, within a nitrogen-filled glovebox, a 20 nm-thick layer of DPPT-TT:PCBM (weight ratio) was deposited onto the substrate by spin coating at 500 rpm for 5 s and then at 2000 rpm for 1 min. This was followed by pre-annealing at 80 °C for 5 min and then annealing at 150 °C for 60 min. PMMA purchased from Sigma Aldrich was dissolved in n-butyl acetate at 80 mg/mL and was spin-coated to serve as the gate dielectric. Spin-coating was performed at a rotational speed of 500 rpm for 5 s and 2000 rpm for 1 min, and the specimens were annealed at 80 °C for 9 h. A 100-nm-thick aluminum gate electrode (Al) was subsequently deposited using thermal evaporation with a shadow mask. Electrical characterizations were conducted in ambient conditions using a probe station coupled to a Keysight B1500A semiconductor parameter analyzer (Keysight Technologies, Colorado Springs, CO, USA). The UV-visible spectra were acquired using the UV-3600 Plus instrument (Shimadzu Corporation, Kyoto, Japan). The Atomic Force Microscopy (AFM) measurements were conducted using the Cypher S AFM instrument (Oxford Instruments-Asylum Research, Santa Barbara, CA, USA). The Raman spectroscopy data were acquired using the MStarter 100 instrument (Metatest Corpotation, Nanjing, China).

## 3. Results and Discussion

When a transistor operates in the linear regime, its drain current (*I*_D_) follows the equation below:

(1) $$I_{D}=\frac{W}{L}\mu C_{i}\left[\left(V_{G}-V_{th}\right)\cdot V_{D}-\frac{1}{2}V_{D}^{2}\right],V_{D}\ll V_{G}-V_{th}$$

where *C*_i_ is the gate capacitance per unit area (4.1 nF/cm^2^ tested by FS-Pro’s capacitance-voltage module), *W* and *L* are the channel width and length, respectively; *V*_G_, *V_D_*, and *Vth* are the gate, drain, and threshold voltage, respectively.

The optoelectronic performance was evaluated by using a Keysight B1500A source meter. Figure 1b illustrates the transfer curves (*I*_D_ − *V*_G_) corresponding to the device’s linear and saturation regimes (*L* = 300 μm). When the device is subjected to *V*_D_ voltages of –1 V and –60 V, it is evident that the gate leakage current (*I*_G_) is insignificant. Notably, when subjected to a drain voltage of –1 V, the device exhibits a threshold voltage of roughly –18 V in Equation (1), similar to the device with PCBM in this work (for other channel lengths, see Supplementary Materials). Furthermore, it displays a subthreshold swing of 8.02 V/dec, relatively higher than that of the device with PCBM. The field-effect mobility of the device is around 0.03 cm^2^V⁻^1^s⁻^1^, which is relatively lower than the devices with PCBM.

Figure 1c shows the Raman spectrum of DPPT-TT, consistent with the previous report [16]. There are four main peaks located at 1371 cm^−1^, 1410 cm^−1^, 1435 cm^−1^, and 1511 cm^−1^. Each peak corresponds to C=C stretching, thienothiophene core, C=C stretching thienothiophene core and C-C stretching thiophene ring, C=C/C-C stretching/shrinking on thiophene, C=C stretching in DPP unit. Figure 1d displays the output characteristics (*I*_D_ − *V*_G_) of the phototransistors, detailing their functionality across a *V*_G_ spanning of 0 to –60 V with step of –10 V. There is a noticeable and distinct transition from the linear regime to the saturation regime, which evidences P-type transistor characteristics.

**Figure 2:**
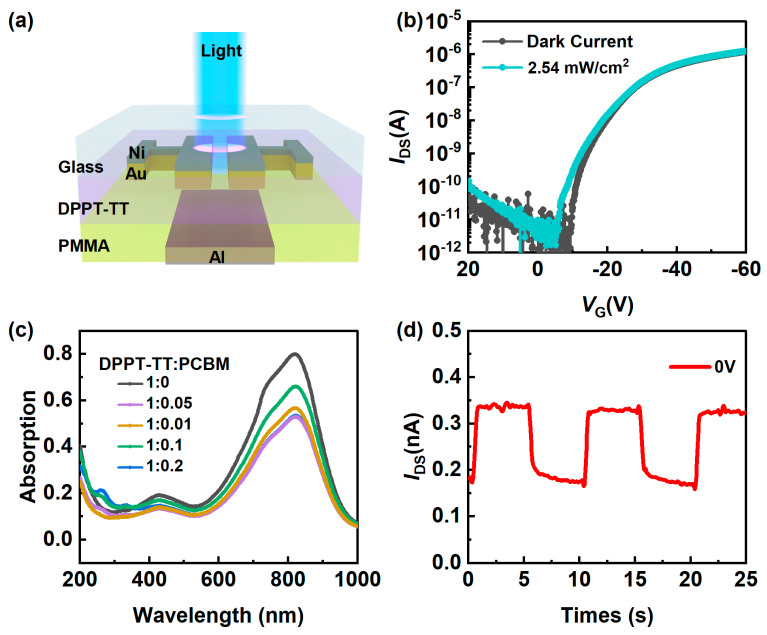
(**a**) Schematic illustration of UV phototransistor measurement. (**b**) Transfer curve of the DPPT-TT phototransistor without PCBM. (**c**) The absorption spectrum of thin films with different ratios of DPPT-TT to PCBM. (**d**) Response time of the DPPT-TT phototransistor without PCBM.

As shown in Figure 2a, the electrical properties of the phototransistors were tested in normal room conditions by using an incident UV light of 365 nm at various intensities to assess UV detection (see Figure S1 for light intensity calibration). The effective photoelectric area was calibrated to be 3.6 × 10^−3^ cm^2^ (here, *W* is 1200 μm and *L* is 300 μm). Note that, upon two distinct light intensities of 0 and 2.54 mW/cm^2^, a very minor shift is observed for the transfer curves, as seen in Figure 2b. The device shows a transition from an “off” to an “on” state at almost the same *V*_G_ of –18 V.

In theory, under UV exposure, photons with energy over the bandgap of DPPT-TT are absorbed, initiating an excitonic transition. This absorption triggers the generation of photoexcited charge carriers, augmenting drain current (*I*_D_) and, thus, pushing the phototransistor’s transition towards the ‘on’ state. As the incident light intensity increases, the rate of photoexcited carrier generation rises proportionally, resulting in a concomitant amplification of *I*_D_. However, owing to the narrow band gap of the semiconductor (1.6 eV for DPPT-TT here [17]), the photogeneration is relatively slight, leading to the swift recombination of the electron–hole pairs that are produced within a brief timeframe. Figure 2c shows the UV-vis absorption spectrum of the thin film comprising the semiconductor layer. For the 365 nm light source, we found that the semiconductor’s absorption of light ranges from 0.108 to 0.195. The slight change in absorption of DPPT-TT in the UV region is mainly because the semiconductor layer is thin [18]. Figure 2d indicates that the rise time of the devices without PCBM nanocrystals is approximately 1 s when *V*_G_ is 0 V.

**Figure 3:**
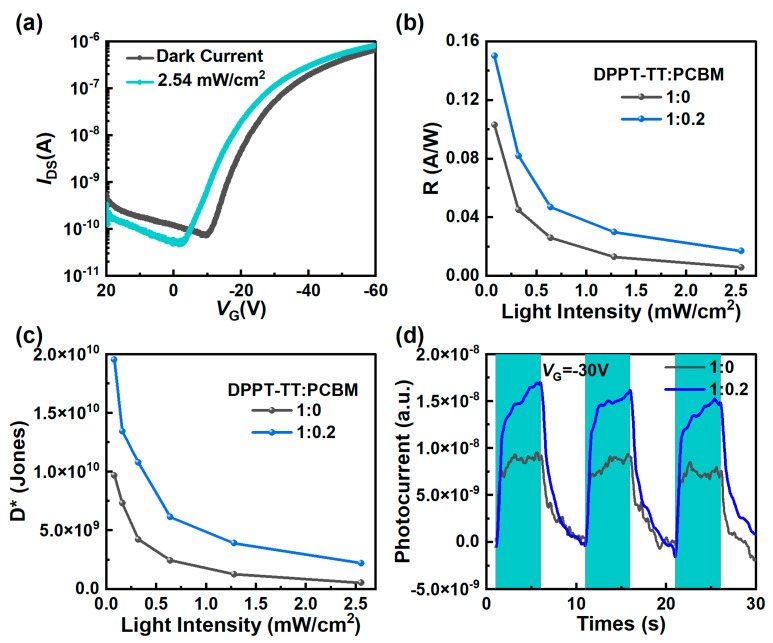
(**a**) Transfer curves of a device with DPPT-TT: PCBM ratio of 1:0.2. (**b**) Responsivity at different light intensities (*V*_D_ = −60 V). (**c**) Specific detectivity with different light intensities (*V*_D_ = −60 V). (**d**) Response time of the device with DPPT-TT:PCBM ratios of 1:0 and 1:0.2.

Responsivity (*R*) and specific detectivity (D*) are essential factors for phototransistors [19]. They can be expressed as:

(2) $$R=\frac{I_{light}-I_{dark}}{S\cdot P}$$

(3) $$D^{*}=\frac{R\cdot \sqrt{S}}{{\left(2q\cdot I_{dark}\right)}^{\frac{1}{2}}}$$

where *I*_light_ is the photocurrent, *I*_dark_ is the dark current, *S* is the effective area, *P* is the light intensity, and *q* is the elementary charge. To enhance the UV photoresponse efficacy of the phototransistors, we systematically varied the PCBM concentration. Figure 3a shows a considerable leftward shift of the transfer curve for the phototransistor with a DPPT-TT:PCBM ratio of 1:0.2, distinct from that observed for the device without PCBM (see Figure 2b). Adding PCBM at that ratio decreases the threshold voltage from –19 to –13 V for a P-type transistor (often applying negative bias). This is mainly due to the introduction of PCBM nanocrystals, which form a heterojunction with DPPT-TT. When UV light is turned on, excitons are generated at the PCBM/DPPT-TT interface. After the electron–hole pairs are separated, some holes are captured by the charge traps in the conducting channel. Therefore, there are trapped charges at the interface between the semiconductor and the dielectric. This leads to an increase in current and a shift in the threshold voltage of the phototransistor. As shown in Figure 3b, the responsivity reaches 0.16 A/W at an optical intensity of 0.08 mW/cm^2^, representing 50% enhancement compared to the devices without nanocrystals. Figure 3c demonstrates that, by adding PCBM, the specific detection rate even doubles, reaching 2.00 × 10^10^ Jones (data of devices with different doping ratios, see Supplementary Materials).

As depicted in Figure 3d, under a light pulse at a frequency of 0.1 Hz and a gate bias *V*_G_ at –30 V, the photocurrent in the presence of light increases by 50% for the devices with PCBM compared to the counterparts without PCBM. Moreover, the change in photocurrent coincides with the frequency of the light pulse. When the light source is turned on, the photocurrent of the device rises rapidly, and the rise time is about 1 s. When the light source is turned off, the photocurrent of the device slowly decreases, which is governed by the recombination of free charge carriers and the trapped charges in polymer material [20,21].

**Figure 4:**
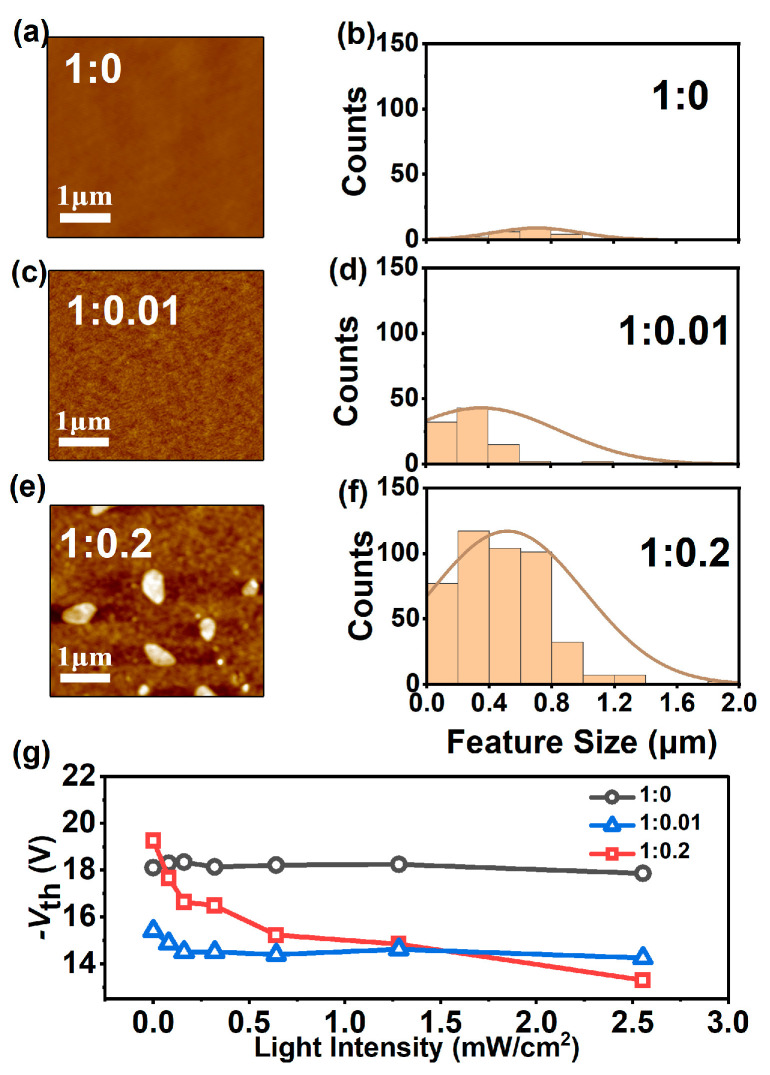
(**a**,**c**,**e**) AFM images of films with different ratios of DPPT-TT to PCBM. (**b**,**d**,**f**) Statistical diagram of feature size of the films with different ratios of DPPT-TT to PCBM. (**g**) Threshold voltage shift of devices under different light intensities with different ratios of DPPT-TT to PCBM.

AFM measurements were carried out on the DPPT-TT films, incorporating different PCBM ratios to gain insight into the PCBM’s microstructural impact on the phototransistors’ electrical properties. The scanning area was set at 5 μm × 5 μm. Figure 4a,c,e illustrate the morphology of the films at three PCBM concentrations. The RMS average values in Figure 4a,c,e are 184.860 pm, 639.867 pm, and 1.730 nm, respectively. Figure 4b,d,f present the corresponding statistics of the feature size and counts. At a concentration ratio of 1:0.2, there is the highest distribution of nanocrystals and the greatest variety in the feature sizes of the nanocrystals.

First, the pure DPPT-TT film is relatively smooth, without noticeable domains or crystals. The threshold voltage of the related phototransistors hardly varies with the incident light intensity, as shown in Figure 4g. By contrast, adding little PCBM (at a ratio of DPPT-TT:PCBM = 1:0.01) sizeably reduces the threshold voltage that is just slightly dependent on the light intensity. The film remains continuous, but microstructures or micro-patterns can be identified. Adding more PCBM up to a ratio of 1:0.2, several crystals appear, with feature sizes as large as 1 μm. Interestingly, the threshold voltage of the corresponding phototransistors shows a strong dependency on the UV light intensity, which is not observed for the other devices with DPPT-TT:PCBM ratios of 1:0 and 1:0.01.

PCBM has long been used as a P-type dopant for organic devices [22]. However, in this work, it is not a traditional dopant but rather a component to form the heterojunction interface between DPPT-TT and PCBM nanocrystals. This is a crucial aspect as it helps form a robust space-charge region, which is vital for effective charge separation and reduced recombination. A ratio of 1:0.01 (DPPT-TT:PCBM) is high enough to induce a doping effect but does not substantially influence the DPPT-TT microstructure. At this ratio, PCBM molecules are impurities in the DPPT-TT and serve as dopants. This doping effect arises spontaneously from thermodynamics, even if in the absence of UV light. A higher ratio of 1:0.2 (DPPT-TT:PCBM) gives rise to a very different scenario. Such a high doping dose causes severe aggregation of PCBM, which is detrimental to doping efficiency. In addition, the molecular self-assembly of DPPT-TT is disrupted by the presence of PCBM nanocrystals. So, the threshold voltage of the relevant phototransistors is even higher than that of the devices with pure DPPT-TT. On the other hand, this configuration with a high ratio of PCBM nanocrystals is beneficial for UV light detection.

**Figure 5:**
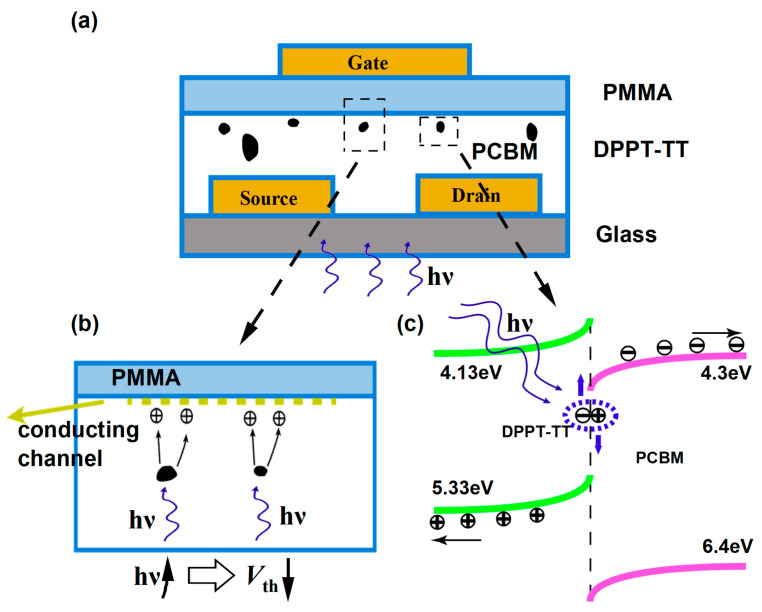
(**a**) Structure and Operational Principles of the Device. (**b**) Schematic diagram of the action of PCBM nanocrystals in the semiconductor layer under UV light. (**c**) Energy band diagram of the nanocrystals region.

In order to investigate the underlying mechanism, Figure 5 provides a schematic representation of the device’s operating principles. Specifically, Figure 5a, Figure 5b, and Figure 5c illustrate the channel conduction and the energy band diagrams at the interface between the DPPT-TT and PCBM nanocrystals, respectively. When a sufficiently high negative gate voltage is applied, holes start to accumulate within the channel. When UV light is turned on, excitons are generated at the interface of DPPT-TT and PCBM nanocrystals. After the electron–hole pairs are effectively separated by the space charge region at the heterojunction interface, some holes continue to enter the conducting channel, as shown in Figure 5b. The existence of excitons at the interface between DPPT-TT and PCBM is depicted in Figure 5c, which suggests that the PCBM nanocrystals embedded in the semiconductor layer create an intermediate state within the band gap. When the semiconductor layer of a phototransistor is exposed to UV radiation, it absorbs UV light. Upon excitation, excitons are indeed formed at the interface. Excitons are free to move, but their mobility is constrained by their lifetime, which limits the distance they can travel. This process is related to the binding energy. An energy level mismatch exists at the interface between the semiconductor layer DPPT-TT and PCBM. It causes the electrons to transfer to the lowest unoccupied molecular orbital (LUMO) with an energy level of 4.3 eV. In the meantime, the holes migrate to the highest occupied molecular orbital (HOMO) with an energy level of 5.33 eV. Under the action of the source-drain electric field, the directed movement of charges is formed, creating electron and hole flow. The mismatch of interface energy levels reduces the electron–hole recombination and concurrently increases the phototransistor’s photogenerated current. The energy level mismatch at the interface between D-A polymer and nanocrystalline particles improves the quantum efficiency, resulting in enhanced UV detection performance of the phototransistors. The presence of an optimal interface, crafted through molecular engineering, reduces the effective barrier for exciton dissociation, enhancing the transfer of electrons to the LUMO and holes to the HOMO.

## 4. Conclusions

In summary, proficiently engineered organic phototransistors for UV detection are presented. This was achieved by integrating PCBM nanocrystals and employing a simplified procedure for device fabrication. Initially, we manufactured a pure D-A polymer phototransistor without PCBM, which had poor detection capability. In contrast, the phototransistor with PCBM nanocrystals exhibited a significant enhancement in current and a noticeable decrease in threshold voltage upon exposure to UV light with an intensity of 2.54 mW/cm^2^. In particular, the photoresponsivity at 1:0.2 ratio of DPPT-TT to PCBM was 0.16 A/W, representing a notable advance of 50% over the device without nanocrystals. Correspondingly, the detection rate reached a value of roughly 2.00 × 10^10^ Jones, exhibiting a twofold increase compared to the device without nanocrystals. This result originates from the feature size and quantity of PCBM nanocrystals in DPPT-TT. This causes a misalignment of energy bands at the interface between PCBM nanocrystals and DPPT-TT, which are responsible for the enhanced photodetection capabilities. This phenomenon leads to an enhancement in the quantum efficiency of the device for ultraviolet light. Therefore, our results demonstrate a straightforward and economically efficient method to optimize organic phototransistors.

## Data Availability

Data are contained within the article.

## Supplementary Materials

The following supporting information can be downloaded at: https://www.mdpi.com/article/10.3390/nano14211748/s1.
