# Supplementary material for: Influence of PCBM Nanocrystals on the Donor-Acceptor Polymer Ultraviolet Phototransistors
Source: Nanomaterials (Basel). 2024 Oct 30;14(21):1748. doi: 10.3390/nano14211748 (PMC11547190; doi:10.3390/nano14211748)
Supplement: Supplementary file 1 [file nanomaterials-14-01748-s001.zip › nanomaterials-3234676-supplementary.pdf]

Supplementary material for

Influence of PCBM Nanocrystals on the Donor-Acceptor Poly-mer Ultraviolet Phototransistors

Hong Zhu [1], Quanhua Chen [1], Lijian Chen [1], Rozalina Binti Zakaria [3], Min-Su Park [4], Chee Leong Tan [1,2], Li Zhu [1,*] and Yong Xu [1,2,*]

1 College of Integrated Circuit Science and Engineering, Nanjing University of Posts and Telecommunications, Nanjing 210023, China; 2020020116@njupt.edu.cn (H.Z.); 2021020307@njupt.edu.cn (Q.C.); 2020020217@njupt.edu.cn (L.C.); cheelong@njupt.edu.cn (C.L.C.)

2 Guangdong Greater Bay Area Institute of Integrated Circuit and System, Guangzhou 510535, China.

3 Photonic Research Centre, University of Malaya, Kuala Lumpur, Malaysia; rozalina@um.edu.my (R.B.Z.)

4 Department of Electronics Engineering, Dong-A University, Busan 49315, Republic of Korea; mpark@dau.ac.kr (M.P.)

* Correspondence: zhuli0319@njupt.edu.cn (L.Z.); xuyong@njupt.edu.cn (Y.X.)

Light source calibration

Light source calibration as shown in figure S1a, the setup for light source calibration (THORLABS) is enclosed within a sealed box to ensure electromagnetic shielding and a light-free environment. Initially, we adjust the vertical distance between the light source and the sensor to 3 cm, which is also the fixed distance between the light source and the device in subsequent testing; see figure S1b. The light source is connected to the signal generator, while the sensor is connected to the power meter. As depicted in Figure S1c, the signal generator on the left adjusts the light power density via voltage control. On the right side, the power meter, connected to the computer host, measures the light power density. After setting up relevant parameters in the software module, the light source calibration begins, and the corresponding light power density and light power can be directly read from the computer screen, as shown in Figure S1d. The relevant parameter data can be found in Table S1.

Table S1. Data from a single light source calibration

AMPL (Vpp)	Irradiance (μW cm^{-2})	Power (μW)
3.242	80.00	7.859
3.338	160.40	15.750
3.480	319.94	31.40
3.702	641.00	62.92
4.060	1280.60	125.73
4.680	2554.00	250.82

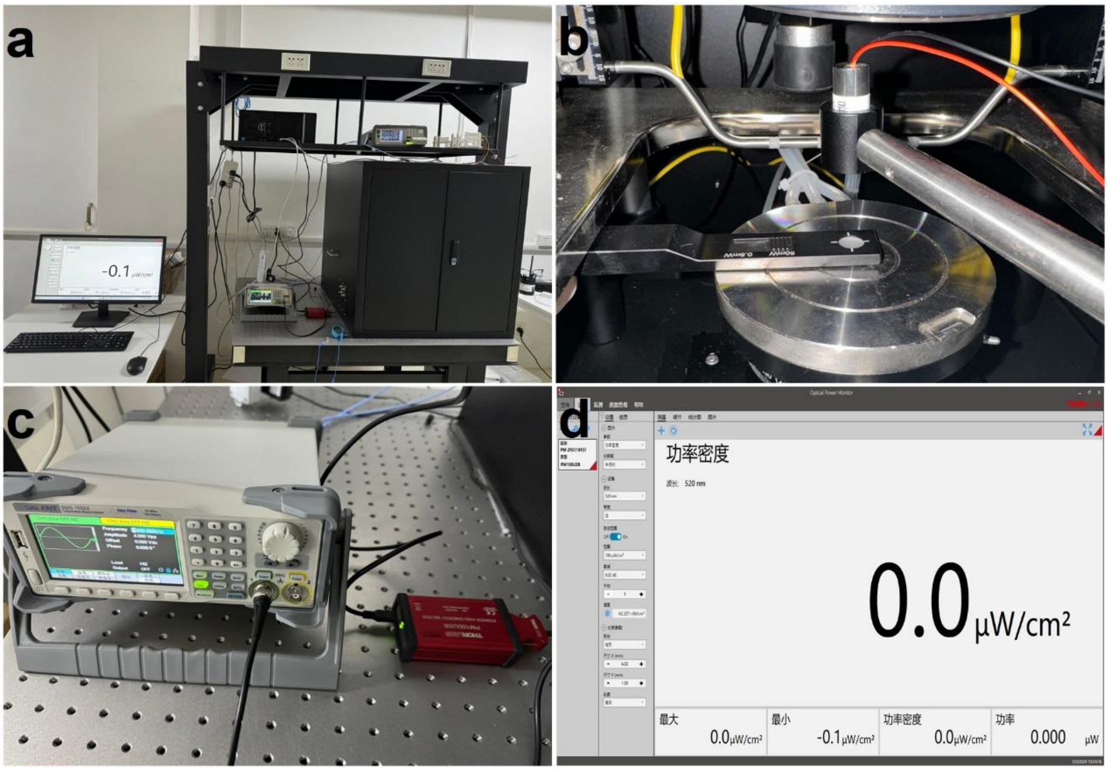

Figure S1. a) Setup of the light source calibration. b) Sensor (down, S130VC) and light source (up, 365nm). c) Signal generator (left, SDG-1032X) and power and energy meter (right, PM100USB). d) Software module of THORLBAS.

The data of transfer and output characteristics for the remaining channel lengths (*L* =150um,

and 500um)

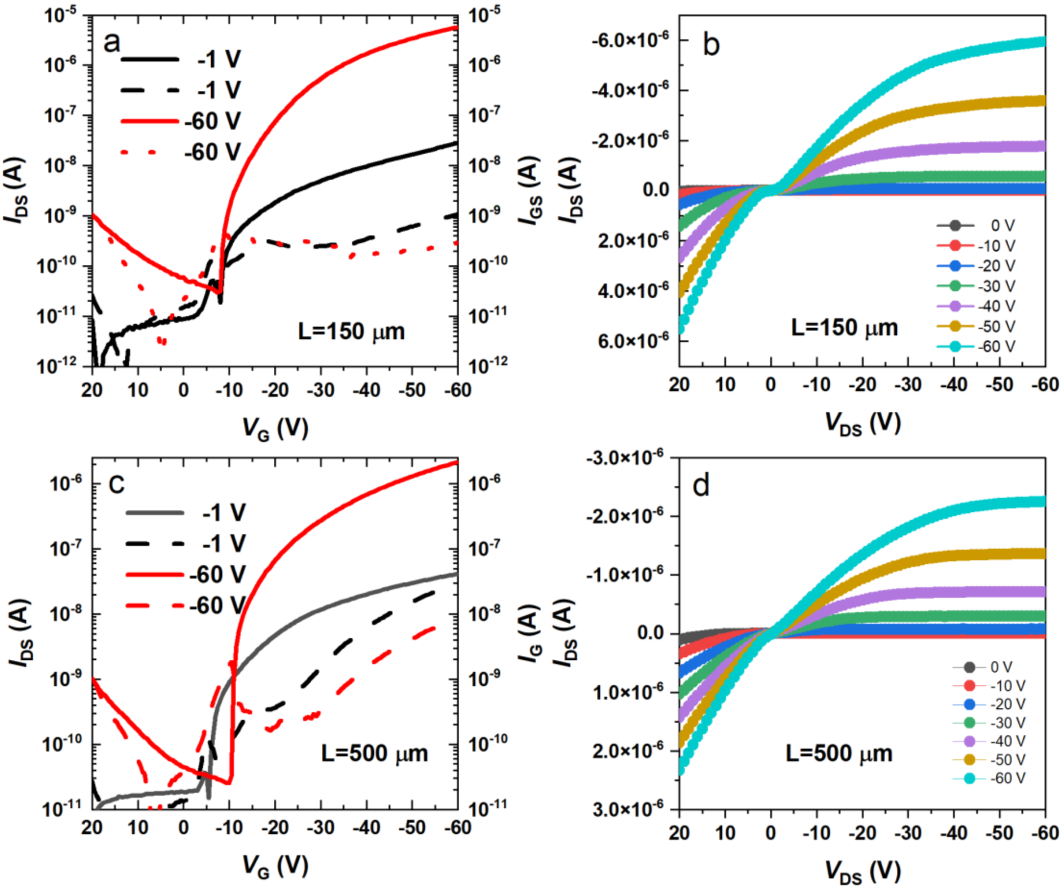

Figures S2. The transfer characteristics (a) and output characteristics (b) with a channel length of 150 microns; the transfer characteristics (c) and output characteristics (d) with a channel length of 500 microns.

The data of transfer characteristics for under various light-intensity densities for different ratios.

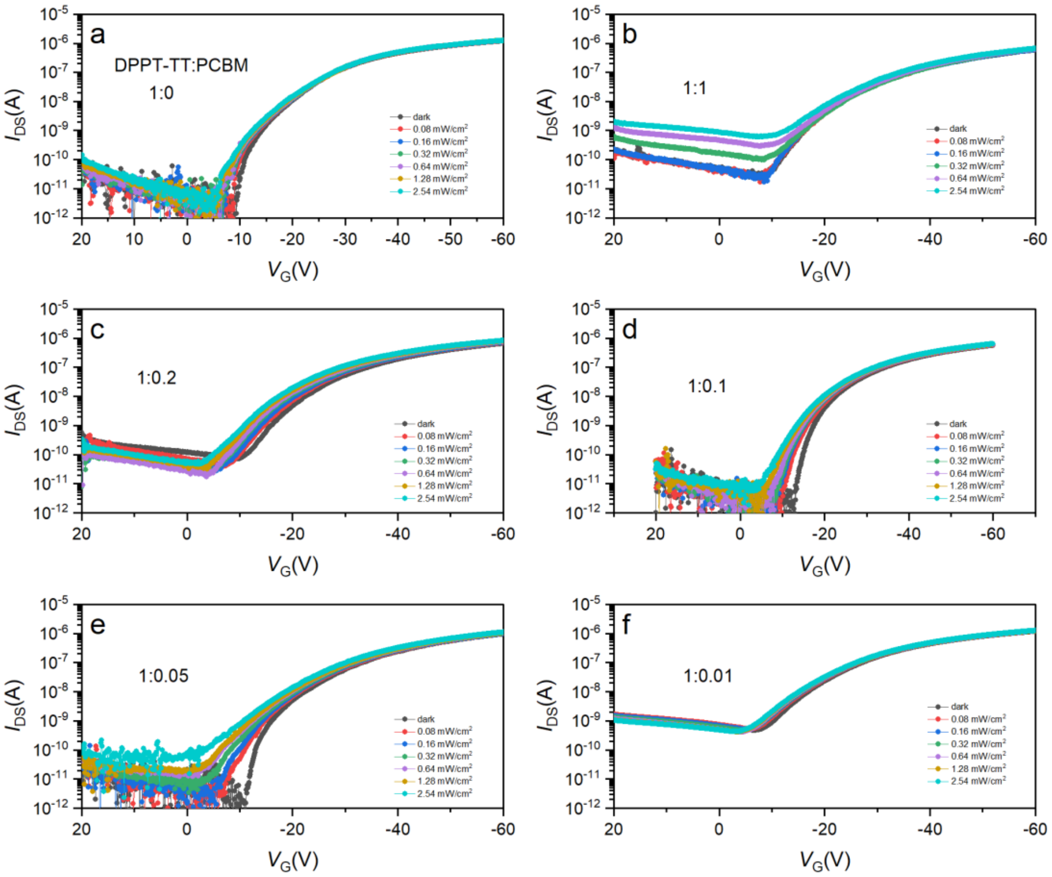

Figures S3. The transfer characteristics for under various light-intensity densities for different DPPT-TT: PCBM ratios. (a) 1: 0, (b) 1: 1, (c) 1: 0.2, (d) 1: 0.1, (e) 1: 0.05, and (f) 1:0.01

The data of output characteristics for different ratios.

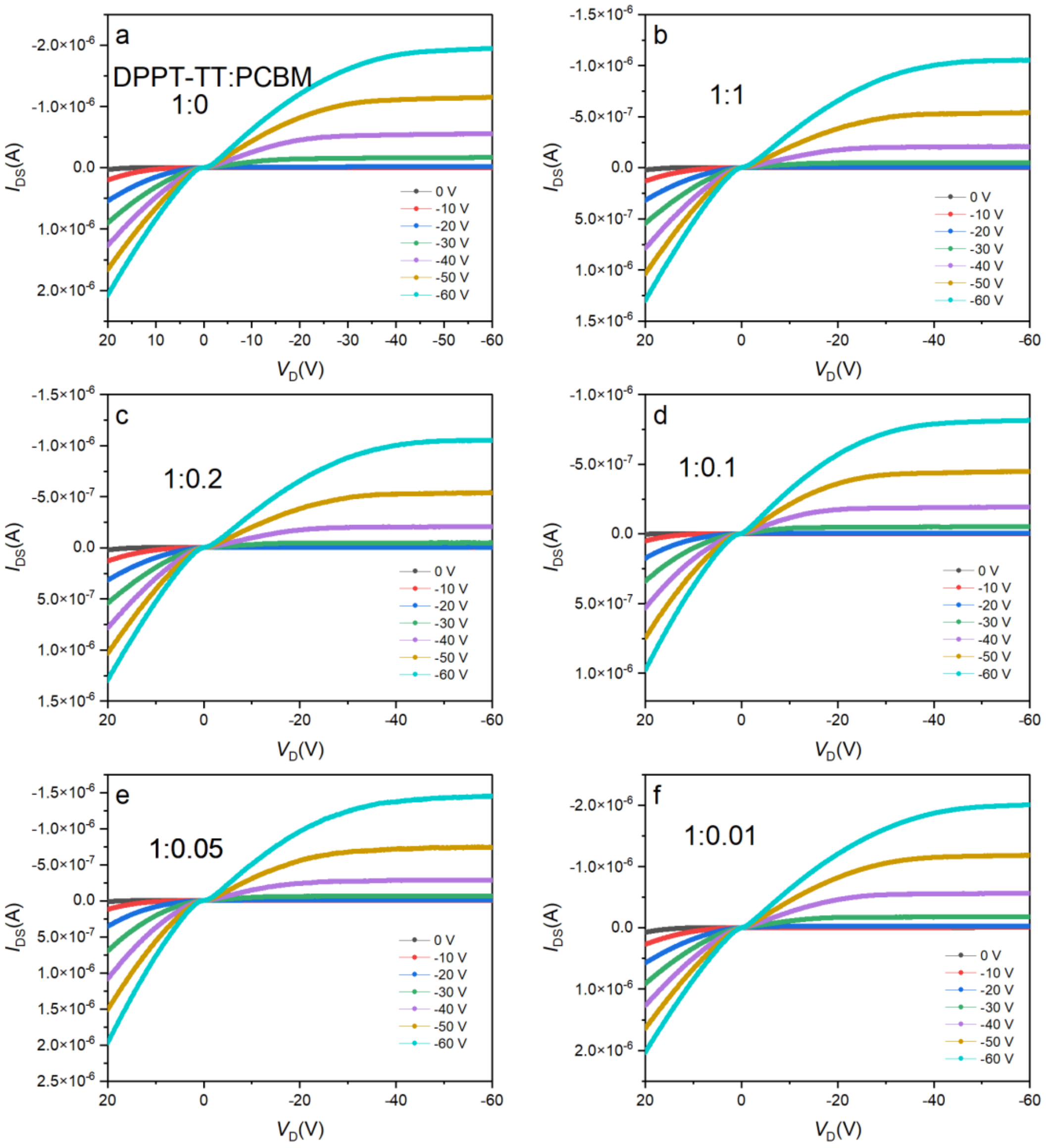

Figures S4. The output characteristics for different DPPT-TT: PCBM ratios. (a) 1: 0, (b) 1: 1, (c) 1: 0.2, (d) 1: 0.1, (e) 1: 0.05, and (f) 1:0.01

AFM images

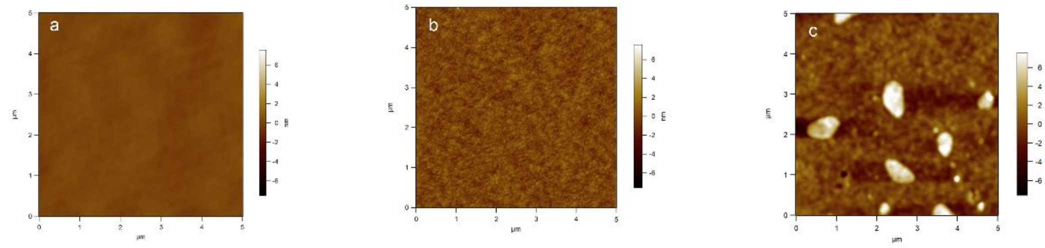

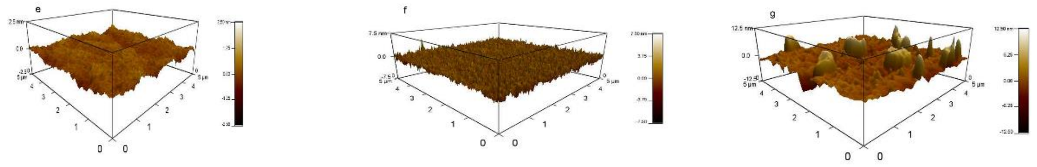

Figure S5. AFM images and 3D mode with different DPPT-TT: PCBM ratios. DPPT-TT: PCBM is 1:0 in a) and e). DPPT-TT: PCBM is 1:0.01 in b) and f). DPPT-TT: PCBM is 1:0.2 in c) and g).

The data of response time for different ratios.

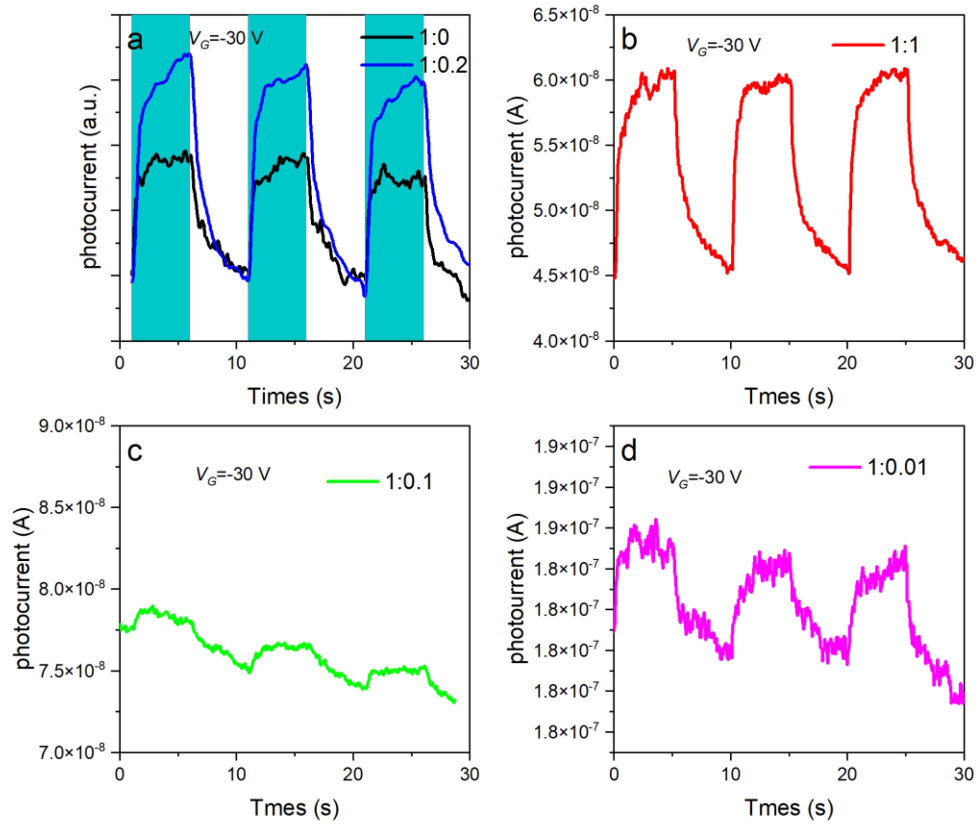

Figure S6. Response time of the device with different DPPT-TT:PCBM ratios

The data of the Raman spectrum for different ratios.

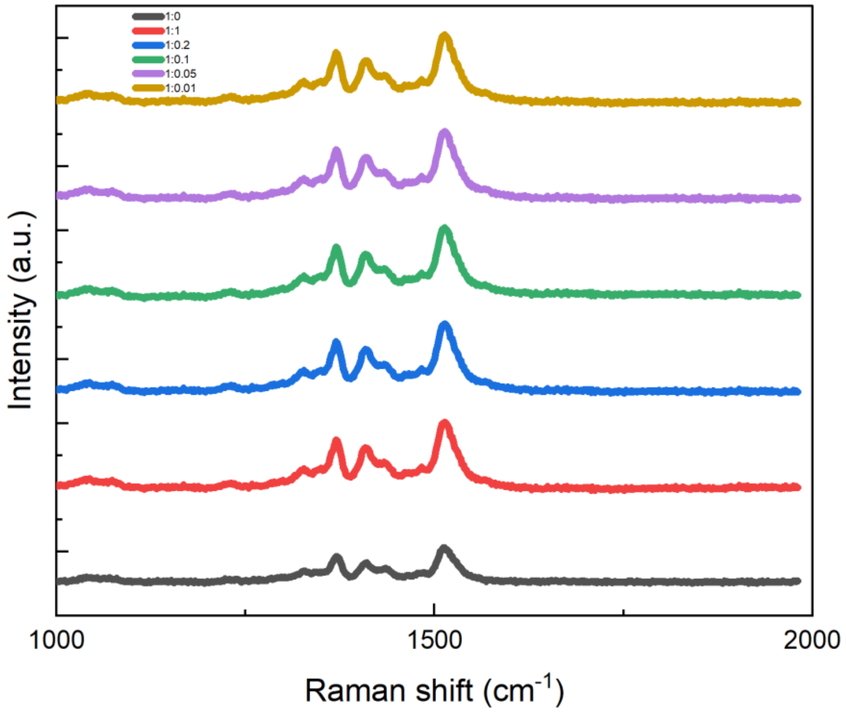

Figure S7. the Raman spectrum of semiconductor layer (DPPT-TT: PCBM) with different ratios
